# Unmasked: an insight into three patients’ rare disease experiences during the COVID-19 pandemic

**DOI:** 10.1186/s13023-021-01734-3

**Published:** 2021-02-26

**Authors:** Catriona Chaplin

**Affiliations:** Barts and the London School of Medicine and Dentistry, Turner St, Whitechapel, London, E1 2AD UK

**Keywords:** Mastocytosis, Covid-19, Rare disease

## Abstract

This article describes my reflections of speaking with three patients and their families living with mastocytosis, who I was introduced to through the UK Mastocytosis Support Group. I discuss the various ways in which the condition affects their day-to-day lives and how this has changed during the Covid-19 pandemic. I have tried to give an insight into the particular difficulties that this patient group faces, both during and before the pandemic, whilst also considering how these challenges may resonate more widely with other patient groups in the rare disease community. Pseudonyms are used throughout to protect patient anonymity.

Limited research, a lack of curative treatment and a lot of uncertainty—in these aspects, Covid-19 bears resemblance to challenges patients with a rare disease have long had to face. Whilst no individual has been untouched by the pandemic, for those with a rare disease, it has presented new difficulties and exacerbated existing problems. Through the UK Mastocytosis Support Group, I had the privilege of speaking with three patients and their families about their experiences of mastocytosis, both before and during the pandemic. I spoke with Beth, a long-standing patient advocate, Hannah, a young charity-worker, and Jane, mother of Emily—who was diagnosed at just 6 months. As they recounted their stories, the diversity of experiences that exists, even within a single patient group, became strikingly apparent. I hope my reflections on these patients’ narratives illuminate some of the issues facing rare disease patients, so that they might be better recognised and addressed as we emerge from the pandemic.

## What is mastocytosis?

Mastocytosis is a chronic condition resulting from an increased number of mast cells, a type of immune cell, in the body. It is extremely rare, affecting 1 in 10,000 [1], meaning patients are often far more knowledgeable than doctors—*“I never take for gospel what a doctor tells me”* Jane informed me. The disease may be limited to the skin, causing itchy rashes and flushing, whilst systemic disease has additional symptoms of severe fatigue, diarrhoea, headaches and abdominal/bone pain, to name a few. These symptoms may be mild, severe or even fatal. Most concerning are sudden, unpredictable episodes of anaphylaxis which, like other symptoms, may occur spontaneously or in response to triggers including temperature extremes, foods, odours, chemicals, stress, infections and medications [2]. “It is the unpredictability of the condition that is most challenging” Beth emphasised. Such uncertainty regarding symptom onset and intensity contributes to the significant impact mastocytosis has on patients’ quality of life [3].

## The impacts of shielding

In March 2020, the government announced 2.2 million people identified as extremely vulnerable would receive letters advising them to ‘shield’ for a minimum of 12 weeks. Hannah received such a letter and so, from mid-March until August, stayed at home bar a few late-night walks. I was interested to know how Hannah felt on receiving this letter, expecting her to lament being unable to meet friends or go for a meal. Her response was much more poignant. “I had never previously thought of myself as vulnerable” she said, describing coming to terms with a new imposed identity centred on her health. Being close in age, I contemplated the difficulty of reassessing one’s sense-of-self, particularly when the media portrayal of ‘shielders’ as frail elderly patients is so divorced from your own reality. The label of ‘shielder’ has very real impacts on identity, creating a sense of ‘otherness’ which may exacerbate feelings of isolation—already common amongst individuals with a rare condition [4]. Indeed, minimal support or follow-up accompanying Hannah’s letter had left her feeling alone, particularly given the gravity of news being delivered. “I had to process the idea that if I go outside I might die” she said, a feeling which contributed to the development of health anxiety. Jane also described the toll of lockdown on Emily’s physical and mental health, observing a “drastic difference in her since going back to school”. Hannah and Emily are not alone; 37% of people shielding saw a deterioration in their mental health [5]. This is particularly concerning given the recognised mental health impact of having a rare condition [6].

Anxiety and isolation have been compounded by uncertainty. Hannah initially received conflicting advice from doctors regarding her shielding status and felt let down by poor clarification regarding her risk. Unfortunately, patients with rare diseases have generally had little explanation as to how their risk has been determined [7]. Given such patients are often managed by multiple specialists [4], there is a greater chance of them receiving contradictory advice—an experience that could be minimised with improved implementation of coordinated care plans.

The consequences of shielding extend far beyond defining a person’s physical reality, having notable impacts on individuals’ identity and mental health. It has also brought to the fore many of the ‘normal’ challenges patients with rare conditions face: isolation, anxiety, weak support structures and poor access to reliable information. Patient organisations like UK Mastocytosis Support Group often fill the gap for many of these problems but, although the former does not appear in jeopardy, many charities have reported substantial funding losses [8] that will have serious knock-on consequences for patients.

## Disruption—medicines, healthcare and food

As the world scrambles to control Covid-19, associated supply-chain disruption and drug-repurposing has led to medication shortages [9]. Jane could not readily obtain famotidine over the summer, a medication without which Emily’s “reactions are uncontrollable, her skin flares excessive and she can’t control her bowel movements”. Before Covid-19, demand for famotidine increased after a similar drug, ranitidine, came off the market. However, studies citing the potential use of famotidine in Covid-19 [10–12] are likely to have exacerbated these shortages. Having felt frustrated at simply being unable to buy paracetamol, I cannot imagine the much greater anxiety and stress Jane experienced—a problem not atypical, with 20% of rare disease patients having difficulty accessing medications during the pandemic [7]. Whilst Covid-related medication shortages have captured media headlines, they are simply another ‘bump in the road’ in on-going medication scarcities and funding restrictions affecting drug availability for rare disease patients.

Disruption to health services and health-professional availability has also, sadly, been the norm. Although the type of disruption will vary between patient groups depending on their healthcare needs, two-thirds of rare disease patients have been affected in some capacity [7]. For mastocytosis patients, Beth described how disruption and poor follow-up has manifested in the late diagnosis of health conditions unrelated to mastocytosis, with clinicians too quickly coupling new symptoms to the patients’ rare condition.

Covid-19 has also led to disturbances in food supplies. With specific dietary requirements due to her triggers, stock-piling behaviour has made it challenging for Beth to access safe, appropriate foods. Mastocytosis patients already have concerns about obtaining adequate nutrition [13], making the impacts of supply shortages on this patient group substantial.

## Adjusting to the ‘new normal’

*“We’re constantly on guard for any potential triggers. This includes going to visit family and ensuring there’s no air fresheners, scented candles or perfumes”* Jane.

Before speaking with Beth, Jane and Hannah, I had failed to appreciate the challenges associated with returning to the ‘new normal’. Universal implementation of rigorous hygiene practices increases the risk of encountering triggers in the form of cleaning and sanitising products. Even before Covid-19, Beth described moving train carriages to avoid “heavily perfumed individuals” triggering her symptoms, including potential anaphylaxis. Widespread, frequent use of cleaning products thus poses a major obstacle to re-entering public spaces. Jane listed numerous, detailed protocols put in place for Emily, who is sensitive to scented cleaning products, to return to school safely. The need for such protocols means Emily is currently unable to visit ‘uncontrolled’ environments outside of home, school and clubs. Concern about the virus may thus be secondary to concerns about measures to control it.

Overcoming the challenges presented by Covid-19 is infinitely more difficult when it requires explaining or justifying one’s health needs to others. Jane and Hannah impressed upon me how poor awareness of mastocytosis, amongst medical professionals and the public alike, has negatively affected them. Given her young age and her condition being mostly ‘invisible’, Hannah faced difficulty in explaining to colleagues her reasons for shielding: “if you have asthma then people just get it, but people can’t pronounce my condition let alone understand it”. For those with mastocytosis, Covid-19 presents unique problems requiring personalised solutions. Key to this is support and understanding from others, and help for patients in communicating their needs.

## Silver-linings?

If silver-linings to all this can be found, it is perhaps in the rapid roll out of telemedicine. This can benefit rare disease patients, who often travel long distances, multiple times a year, to visit specialists [4]. With mastocytosis, the gain from reducing unnecessary travel, and thus exposure to triggers on transport or overnight accommodation, is even greater. Virtual clinical trials, stakeholder collaboration and stream-lined drug development have also emerged as opportunities [14]] that should be carried forward beyond the pandemic.

## The road ahead

With teaching moved online, I have spent much of the last 6 months reading lists of symptoms from medical textbooks. This has taught me little about real patient experiences, just as reading newspaper headlines has provided little insight into how the pandemic has really impacted peoples’ day-to-day lives. Speaking with Beth, Jane and Hannah has been invaluable, showing me the unique challenges patients have faced during the pandemic. Yet, I have been struck by how many of these difficulties are ultimately a reflection of on-going problems for rare disease patients. I hope that in highlighting current gaps in support, the pandemic may stir positive change and deliver improved care provision for rare disease patients.

## Data Availability

Not applicable.
